# Optimizing Nitrogen Management in Food and Energy Production and Environmental Protection: Summary Statement from the Second International Nitrogen Conference

**DOI:** 10.1100/tsw.2001.481

**Published:** 2001-12-20

**Authors:** Ellis Cowling, James Galloway, Cari Furiness, Mary Barber, Ton Bresser, Ken Cassman, Jan Willem Erisman, Richard Haeuber, Robert Howarth, Jerry Melillo, William Moomaw, Arvin Mosier, Kaj Sanders, Sybil Seitzinger, Stan Smeulders, Robert Socolow, Daniel Walters, Ford West, Zhaoliang Zhu

**Affiliations:** North Carolina State University, College of Natural Resources, Ralegh, NC 27606, USA

**Keywords:** nitrogen, N, energy production, food production, nitrogen cascade, ecosystem impacts, environmental protection, nitrogen management, ammonia, NO_x_, decision makers, reactive nitrogen, nutrient use efficiency

## Abstract

Human efforts to produce food and energy are changing the nitrogen (N) cycle of the Earth. Many of these changes are highly beneficial for humans, while others are detrimental to people and the environment. These changes transcend scientific disciplines, geographical boundaries, and political structures. They challenge the creative minds of natural and social scientists, economists, engineers, business leaders, and decision makers. The Second International Nitrogen Conference was designed to facilitate communications among all stakeholders in the “nitrogen community” of the world. The Conference participants’ goal in the years and decades ahead is to encourage every country to make optimal choices about N management in food production and consumption, energy production and use, and environmental protection. Scientific findings and recommendations for decision makers that emerged from the Conference are presented.

## I. INTRODUCTION

This report summarizes scientific findings and recommendations for decision makers from the Second International Nitrogen Conference held at the Bolger Center in Potomac, MD on October 14–18, 2001. This Conference was organized as an outgrowth of the First International Nitrogen Conference — "Nitrogen, the Confer-N-s" — held in The Netherlands on March 23–27, 1998. Both conferences were organized under the general auspices of the Convention on Long-Range Transboundary Air Pollution (CLRTAP) of the United Nations Economic Commission for Europe (UNECE).

The primary objectives of the Second Conference were to:

- Increase scientific knowledge about reactive nitrogen (Nr)[1] sources and effects on people and the environment,
- Stimulate communication among leaders involved in N production and consumption, and
- Explore balanced strategies to increase food and energy production while decreasing impacts on people and the environment, thereby making progress toward the general theme of the conference: "Optimizing Nitrogen Management in Food and Energy Production and Environmental Protection".

This summary statement has been organized as follows:

Section II. Scientific Summary Statements
 A. Scale and Scope of Nr
 B. Effects of Increased Nr in the Environment
 C. Food Production and Nr
 D. Energy Production and Nr
Section III. Recommendations for Research and Education
Section IV. Management Recommendations for Decision Makers
Section V. Plans for the Third International Nitrogen Conference
Section VI. Publications from the Second International Nitrogen Conference

## II. SCIENTIFIC SUMMARY STATEMENTS

### A. Scale and Scope of Nr

- N is essential for life. It is a fundamental component of the nucleic acids that determine the genetic character of all living things and the enzymes that drive the metabolic machinery of every living cell.
- Before gaseous N ($N_2$) molecules can be used by organisms, the triple bonds of nonreactive $N_2$ molecules must be broken down to single N atoms. These single atoms become reactive forms of N when they bond with other essential nutrient elements: oxygen, carbon, or hydrogen.
- Before 1860, natural biological N fixation (BNF) was the dominant Nr source for the terrestrial biosphere. The rate of Nr creation was less than the demand in many terrestrial and aquatic ecosystems. The remarkable diversity among plants, animals, insects, and microorganisms found in nature is partially the result of intense competition among life forms — many of which evolved under N-limited conditions.
- Today, human-induced production and release of Nr into the environment is ~160 Tg N/year, about 15 times greater than the human contribution in 1860 and still growing. It dominates terrestrial Nr creation globally. The largest contemporary global Nr sources are production and use of $NH_3$ by the Haber-Bosch process (~100 Tg N/year; mostly for synthetic N fertilizers), widespread planting of N-fixing legumes (~35 Tg N/year), and production of energy through combustion of fossil fuels (~25 Tg N/year).
- There are large regional disparities in Nr creation rates on both absolute and per capita bases. Total Nr creation in Asia is larger than in any other region. Per capita Nr creation is largest in North America and Europe. Humans also redistribute large amounts of Nr from one country or region of the world to another through exports of fertilizers, feed grains, and fossil fuels.
- Most plants, animals, and microorganisms in the biosphere are adapted to efficiently use and retain small increments of additional Nr. Thus, addition of Nr to most ecosystems first leads to increased uptake, storage, and use, and hence to increased food or fiber production. Further additions of Nr beyond an optimal amount lead to imbalances in the N cycle and potential leakages in the form of emissions of different forms of Nr to other compartments (see discussion of the N cascade in section B).
- A large, but as yet unknown, portion of the Nr created every year by human action accumulates in the environment. Thus, the concentration of Nr is increasing in many environmental reservoirs. "Hot spots" of Nr creation and emissions occur on almost every continent — in industrial areas, especially where fossil fuel combustion is intense, and in agricultural areas, especially where confined animal feeding operations are concentrated.
- The only way to remove Nr from circulation is by storage in long-term reservoirs (e.g., soils, sediments, and biomass) or conversion to $N_2$ by denitrification. In some cases, it may be possible to capture Nr emissions and deliver them to food or fiber production areas where there are N deficiencies.
- The population of the world is expected to peak at ~9 billion people at the end of this century. At that time, if all humans have the same per capita Nr creation rate as they do today, then the global Nr creation rate will be ~250 Tg N/year compared to the current ~160 Tg N/year. If all people have the same Nr creation rate as exists in North America today (~100 kg N/person/year), then the global rate will be ~900 Tg N/year.

[1] The term reactive nitrogen (Nr) as used in this Summary Statement includes all **biologically active, photochemically reactive,** and **radiatively active N compounds** in the atmosphere and biosphere of the Earth. Thus, Nr includes inorganic reduced forms of N (e.g., $NH_3$, $NH_4^+$), inorganic oxidized forms (e.g., $NO_x$, $HNO_3$, $N_2O$, $NO_3^-$), and organic compounds (e.g., urea, amines, proteins).

### B. Effects of Increased Nr in the Environment

Human-induced increases of Nr contribute to a wide variety of sometimes beneficial and sometimes detrimental changes in the health and welfare of people and ecosystems.

Direct effects of Nr on human health include:

- Increased yields and nutritional quality of the foods needed to meet dietary requirements and food preferences for growing populations
- Respiratory and cardiac disease induced by exposure to high concentrations of ozone and fine particulate matter
- Nitrate and nitrite contamination of drinking water leading to the “blue-baby syndrome”

Direct effects of Nr on ecosystems include:

- Increased productivity of Nr-limited natural ecosystems
- Ozone-induced injury to crop, forest, and natural ecosystems, and predisposition to attack by pathogens and insects
- Acidification and eutrophication effects on forests, soils, and freshwater aquatic ecosystems
- Eutrophication and hypoxia in coastal ecosystems
- N saturation of soils in forests and other natural ecosystems
- Biodiversity losses in terrestrial and aquatic ecosystems and invasions by N-loving weeds
- Changes in abundance of beneficial soil organisms that alter ecosystem functions

Indirect effects of Nr on other societal values include:

- Increased wealth and well being of human populations in many parts of the world
- Significant changes in patterns of land use
- Regional hazes that decrease visibility at scenic vistas and airports
- Depletion of stratospheric ozone by $N_2O$ emissions
- Global climate change induced by emissions of $N_2O$ and formation of tropospheric ozone
- Damage to useful materials and cultural artifacts by ozone, other oxidants, and acid deposition
- Long-distance transport of Nr, which causes harmful effects in countries distant from emission sources and/or increased background concentrations of ozone and fine particulate matter

In addition to these effects, it is important to recognize that:

- The magnitude of Nr flux often determines whether effects are beneficial or detrimental,
- All of these effects are linked by biogeochemical circulation pathways of Nr,
- Nr is easily transformed among reduced and oxidized forms in many systems, and
- Nr is easily distributed by hydrologic and atmospheric transport processes.

Thus, a single Nr molecule can cascade through a wide variety of environmental systems and contribute to multiple sequential effects, as shown in the simplified schematic diagram in Figure 1.

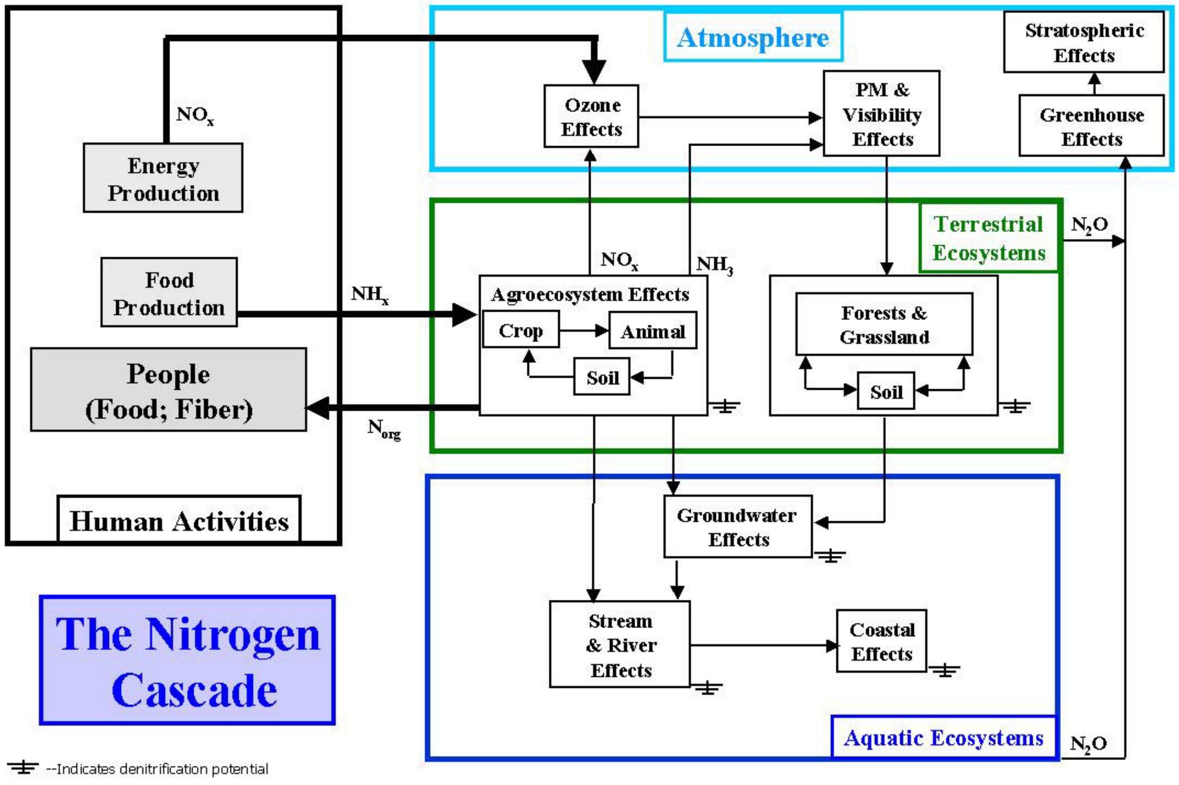

**FIGURE 1.** The N cascade illustrates the movement of human-produced reactive nitrogen (Nr) as it cycles through environmental reservoirs in the atmosphere, terrestrial ecosystems, and aquatic ecosystems (from plenary presentation by J. Galloway).

### C. Food Production and Nr

- Farmers have always explored ways to increase food production per unit area of land. From earliest times, they recycled nutrients in animal and human wastes. Later they imported guano as a N source. Today, farmers in all but the poorest countries depend on synthetic N fertilizers to boost yields.
- About 40% of the present ~6 billion global population is dependent on synthetic N fertilizers produced by the Haber-Bosch process that converts nonreactive $N_2$ into biologically active $NH_3$.
- Food production must increase substantially in the decades ahead to meet dietary needs and food preferences of a larger and wealthier global population. Historically, preferences for animal protein in human diets have increased with every increment of per capita income. At present, about one third of world grain production is used as feed for meat animals. In most of the world, livestock will contribute an increasing fraction of the N in human diets.
- Increased food and fiber production should come primarily from existing arable land through intensification of cultivation so as to protect natural ecosystems and biodiversity. Because little additional arable land is available, site-specific precision agriculture approaches that minimize fertilizer use must be employed where feasible to produce optimum yields. This is especially necessary in high-nutrient–consuming crops including the major cereal grains of the world — maize, rice, and wheat (see Figure 2).
- Even well-managed agricultural lands lose a substantial fraction of their fertilizer Nr inputs. Once lost, the released Nr can cascade through ecosystems, where it alters their dynamics and in many cases demonstrably reduces their ability to supply ecosystem services. In addition, it results in substantial increases in emissions of $N_2O$ that contribute to global warming and stratospheric ozone depletion.
- Forage and feed grain N-use efficiency of food animals vary greatly among ruminants (cattle, goats, and sheep) and nonruminant food animals (swine, poultry, and fish). Typical rates of on-farm N-use efficiency for production of human-digestible protein from feed grains and forages are ~50–60% for fish, ~40–50% for poultry and eggs, ~35–40% for dairy, ~30–40% for swine, and ~15–30% for beef. The remaining fraction (~40–85% of feed-grain N) can be re-used as manure-based fertilizer, but often is lost as air emissions of ammonia or by leaching of nitrate to ground or surface waters.
- Therefore, farmers, foresters, and aquaculturalists must make significant improvements in N-use efficiency to achieve the crop-, forest-, and animal-agricultural yields needed to feed, clothe, and house the world's people, while sustaining environmental quality within and beyond managed landscapes.
- Improving N-use efficiency in major food crops will not be easy. It will require collaboration among ecologists, agronomists, soil scientists, agricultural economists, and politicians. Great needs exist for accurate measurements of actual fertilizer N-use efficiency, N losses, and loss pathways in major cropping systems. Only in this way can we: (1) identify opportunities for increased N-use efficiency by improved crop and soil management; (2) quantify N-loss pathways in major food crops; and (3) improve human understanding of local, regional, and global N balances and N losses from major cropping systems. The starting point for any improvement has to be a clear understanding of the fluxes and balances of N at the farm level. Direct on-farm measurements are necessary because estimates from small plots on research stations overestimate field-scale fertilizer N-use efficiency.
- Livestock production systems offer large opportunities for improvement of N-use efficiency. Where livestock are fed N-fertilized grain (mostly in North America, Europe, and China), changes in feed composition can increase N-use efficiency in food animals without affecting the quality of human-digestible protein in meat products.
- In recent decades, powerful economic and social forces, including economies of scale, efficiency of specialization,

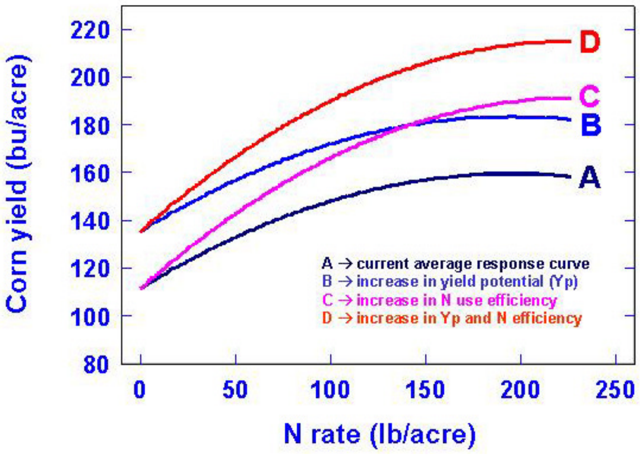

**FIGURE 2.** Current average response of corn yield to N fertilization rate, shown with response after potential increases in yield potential, N-use efficiency, and both (from plenary presentation by K. Cassman).

cheap food and transportation policies, and global competitiveness, have transformed livestock and meat-processing industries in many countries. These changes often lead to largely unforeseen Nr-induced environmental problems mainly on local and regional scales:

1. **Intensification** in confined animal feeding operations increases ammonia emissions and nitrate discharges.
2. **Decoupling,** the physical separation of the land where feed grains are produced from the site on which food animals are raised, decreases recycling of animal manures.
3. **Regionalization and globalization** of markets increase $NO_x$ from transport vehicles as feed, animals, manures, and finished products are transported — often from Nr-poor to Nr-rich areas.
4. **Vertical integration** has great potential to maximize N-use efficiency with integrated economic and advisory-service linkages among farmers, feed suppliers, animal-rearing advisors, and food-processing companies. However, emphasis on economic efficiency without attention to Nr-induced health and environmental risks leads to externalization rather than internalization of these real costs.

- Farmers must show a profit to continue farming. Because maximization of profits is generally not congruent with minimizing losses of Nr from agriculture, optimization of systems to meet these objectives (and others) will be required. Optimization can best be achieved if the external costs of N losses from agriculture are internalized. This means that consumers must pay more for their food. Costs that should be included in the price of food are the costs for production and the farmer's profit, but also the environmental costs associated with fertilizer production and transport, transport of resources and products, and waste processing, as well as the costs associated with decreases in environmental goods and services.
- Policies designed to promote greater N-use efficiency in agriculture should emphasize incentives to farmers (i.e., paying farmers to be good stewards) rather than punitive regulations, so as to avoid export of crop and livestock production to areas with less stringent environmental guidelines.
- It is important to restore and maintain both carbon and N pools in agricultural soils, while at the same time sustaining yield increases to meet food demand and achieving substantial increases in the efficiency with which applied N inputs from both inorganic and organic sources are utilized. Indeed, maintaining soil quality, sustaining yield increases, and minimizing N losses are some of the greatest scientific challenges confronting efforts to address the global N problem.
- Overconsumption of protein-rich foods and consequent excretion of urea by humans is a growing source of Nr. This Nr is presently processed in municipal sewage treatment systems that are rarely designed to facilitate capture of available Nr for use in agriculture and forest production.

### D. Energy Production and Nr

- Combustion of fossil fuels forms $NO_x$ as a waste product from fuel-N (organic N) and atmospheric-N ($N_2$). The primary sources of $NO_x$ emissions are combustion of coal, oil, and natural gas for energy production and use (e.g., generation of electricity, transportation, industrial and construction processes, domestic space and water heating, etc.).
- Global energy consumption is projected to increase ~2–3% annually from 1999 to 2020, for a total increase of ~60% over current rates. The bulk of this increase will occur in developing countries. For example, China's energy consumption is expected to triple by 2020, with energy consumption expected to increase by ~4.5 %/year in all of Asia (Figure 3). In the industrialized world, in contrast, growth in energy consumption is estimated at less than 2%/year due to market saturation and advances in energy efficiency.
- Natural gas use is expected to more than double in many industrialized countries. While the energy market share for coal is projected to decline in Europe and Japan, coal is still expected to be the most common fuel for power generation in 2020, with an estimated 31% share. Coal is expected to remain a major source of energy in the developing world, most notably in China and India, where heavy reliance on coal consumption is projected to continue through 2020.
- Over the next 2 decades, transportation fuel use is expected to grow by nearly 5%/year in developing countries, compared to average annual increases of less than 2% in industrialized countries. Transportation energy use in large parts of Asia is projected to increase by ~7%/year between 1999 and 2020. Much of this growth is expected to be in the "on-road" sector — a combination of freight movement and personal motor vehicle use. Personal vehicle ownership is seen as a symbol of prosperity, and annual car sales are growing rapidly in many Asian countries.
- Trends in the growth of fossil fuel use are alarming given the associated increase in air pollution and impacts on human health and the environment. Currently, the transportation and electric generation sectors are the dominant contributors to $NO_x$ emissions in North America, Europe, and Asia. While $NO_x$ emission trends in North America and Europe are projected to decline in the future due to regulatory measures, global $NO_x$ emissions will probably increase as developing countries increase their standard of living by consuming more electricity and driving more.
- Once N is emitted to the atmosphere as $NO_x$, it can cascade through the environment and contribute to smog, fine particle formation, visibility impairment, acid deposition, excess nutrient inputs to estuaries and near-coastal waters, global warming, and stratospheric ozone depletion.
- These phenomena contribute to detrimental effects on human health and the environment. As a precursor to particulate matter and ozone formation, $NO_x$ emissions can lead to premature death, chronic respiratory illness (e.g., bronchitis

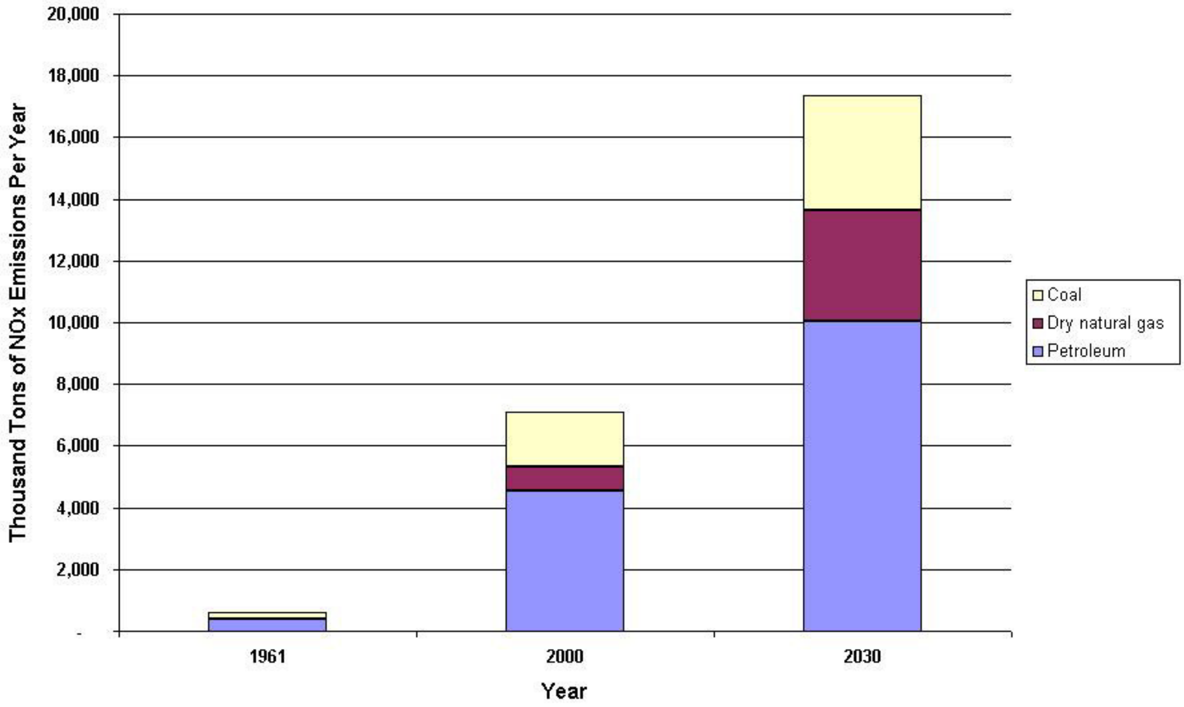

**FIGURE 3.** $NO_x$ emissions in Asia from fossil energy consumption in the years 1961, 2000, and 2030 (from plenary presentation by W. Bradley). Zheng, X., Huang, Y., Xu, X., Wang, Y., Han, S., and Hu, F. (2001) Potential stresses of Asian agricultural activities on the environment: an analysis from nitrogen cycle point of view. *Global Change Biology*, submitted.

or asthma), and aggravation of existing respiratory conditions. Environmental impacts of $NO_x$ emissions and deposition include forest dieback, biodiversity loss in grasslands, acidification of streams and lakes, harmful algal blooms in coastal waters, and global warming. In the U.S., these human health and environmental impacts are found in many areas of the country (e.g., both eastern and western states), and cost society tens of billions of dollars each year when taken together.

- The effects of $NO_x$ on human health and ecosystems are of sufficient magnitude to result in regulations in many countries. $NO_x$ emissions regulations vary greatly from one country to another, but regulated source categories generally include stationary sources (e.g., power generators and industrial boilers) and mobile sources (e.g., automobiles, trucks, and construction machinery).
- On a global basis, energy production has not contributed to total Nr increases to the same extent as N use in agriculture, but there are many current examples of the technical capability to decrease $NO_x$ emissions from fossil fuel burning sources worldwide. Over the last 30 years, technological advances have achieved significant decreases in $NO_x$ emission rates from both mobile sources (emissions per km or mile traveled) and stationary sources (emissions per kWh). Engineering solutions currently exist to decrease most of the $NO_x$ emissions from power generation and mobile sources and, in many cases, can be exported to other countries with few variations.
- Despite declining emissions rates, however, total $NO_x$ emissions have remained level or even increased over the same period in North America and Europe due to increases in vehicle km or mile traveled, electricity usage, and the sometimes-differing regulatory frameworks applied to various sectors. Decreasing total $NO_x$ emissions likely means that continuing technological advances may need to be combined with regulatory approaches (e.g., emissions caps).
- It is now technically feasible and likely economically possible to further decrease $NO_x$ emissions from fossil fuel combustion to the point where they become only a minor disturbance to the N cycle at all scales. Clean electric generation and transportation technologies are commercially available today, or will be commercially available within 1 to 2 decades, which have the potential to further decrease $NO_x$ emissions in industrialized countries and to decrease $NO_x$ emissions from projected business-as-usual levels in developing countries. In addition, technologies currently under development, such as renewable energy and hydrocarbon-based fuel cells, could operate with zero $NO_x$ emissions.
- The vast majority of the world's population lives in regions where decreases in $NO_x$ emissions have not occurred. Indeed, in many countries, significant increases in $NO_x$ emissions are projected to occur over the next several decades due to both population growth and per capita increases in fossil fuel use. The biggest opportunity for decreases in $NO_x$ emissions in the developing world involves a "technology leap" through adoption of advanced technologies such as zero emission-distributed power (e.g., photovoltaic, wind, small hydro, and fuel cells) and near-zero and zero emission electric propulsion vehicles (e.g., electric, hybrid electric, and fuel cells).

### III. RECOMMENDATIONS FOR RESEARCH AND EDUCATION

- Foster multidisciplinary innovations in the management of N, e.g., (1) research by agricultural economists on possibilities for internalization of environmental costs in crop and animal agriculture production systems, and (2) research by agronomists and agricultural engineers on means by which to integrate animal manures more effectively into crop nutrient management plans.
- Determine and prepare maps of critical Nr loads for the atmosphere, terrestrial, and aquatic ecosystems, below which no unwanted effects occur.
- Improve scientific understanding of gaseous emissions of ammonia and other Nr compounds, atmospheric transport and transformation processes at all scales from local to global, and wet and dry deposition processes.
- Improve scientific understanding of the rates of $N_2O$ emission in terrestrial and aquatic ecosystems and the relationships between Nr creation and $N_2O$ emissions.
- Increase scientific knowledge of the fate and residence time of Nr in various parts of the N cascade through accumulation in soils, sediments, and biomass, and improve quantification of Nr flows between stages within the cascade.
- Investigate factors that regulate plant and microbial processing of Nr in natural and managed “sinks” for N in landscapes. These sinks include riparian buffer zones, in- and near-stream wetlands, and in-stream processes that remove Nr from upland areas.
- Determine at different spatial scales (plot, field, watershed, regional) the rates and factors controlling microbial processes by which Nr is or can be denitrified to nonreactive $N_2$ from each reservoir in the N cascade (Figure 1).
- Determine how much of organic-N sources and fertilizer-applied N is retained in the human-digestible portion of crops, in crop and forest residues and humus, in harvested timber and other fiber products, and as additions to the endogenous N reservoirs in agricultural and forest soils, with emphasis on obtaining more accurate estimates under actual production conditions for both crop and forest production systems.
- Evaluate alternative cropping and domestic animal rearing systems, including traditional practices, not only for their impact on current farm productivity and profitability, but also on both short- and long-term N-use efficiency, on rates of loss of both endogenous and fertilizer sources of N, and on associated soil quality traits that govern future productive capacity and ecosystem capacity to retain Nr and minimize losses.
- Answer the “nitrogen legacy question” by determining the extent to which Nr accumulated in various ecosystem reservoirs in recent decades is retained and how these accumulations of Nr will affect the future productivity, stability, and resiliency of crops, forests, and natural ecosystems.
- Determine the technological and economic feasibility, social acceptability, and environmental sustainability of innovative on-farm and centralized systems for converting animal manures and various other types of commercial, industrial, and municipal N-waste streams into value-added products that can be sold at a profit.
- Focus new initiatives in research on options that will reuse or remove Nr before it cascades through the environment. Especially promising target areas include: (1) decreasing $NO_x$ emissions from fossil-fuel combustion in power plants and industry by precombustion removal of organic N, (2) converting $NO_x$ in flue gas streams into saleable fertilizer products, (3) increasing N-use efficiency by crop and animal production systems, (4) decreasing Nr losses from animal wastes, and (5) recycling Nr back on the land from which feed grains and forages are produced.
- Further develop integrated assessment models, such as NitroGenius, to (1) explore different cost-effective options to diminish the cascade of effects of Nr, and (2) communicate the integrated nature of the N cascade to both the scientific (students and researchers) and policy communities.
- Further develop cause-effect relationships and magnitude of response to decreases in Nr inputs so as to support characterization, and as appropriate, monetization of environmental benefits.
- Develop integrated research approaches that address Nr issues in the context of linkages with other nutrient cycles, especially carbon, sulfur, and phosphorus.
- Develop multipollutant, multieffect strategies to optimally combat environmental effects resulting from human activities. Develop technological road maps for future infrastructures where fossil fuels can be replaced by renewable energy sources, e.g., through exploration of hydrogen-based rather than fossil-fuel–based energy production systems.
- Work to include the developing world more fully in the N knowledge base regarding impacts and available solutions. Establish an institutional framework that supports exchange of information between researchers on the effects of Nr in its various aspects, e.g., food production, use of fossil fuels, and the environment; and identify mechanisms, such as the World Bank, to fund researchers in the developing world to investigate specific regional impacts and solutions.
- Focus new initiatives in education on established, but underutilized, site-specific best management practices (BMPs) known to improve N-use efficiency in crop and animal production systems. Examples include timing of N applications to meet crop uptake, balanced crop nutrition, and appropriate rates of manure application.
- Include more emphasis in medical and public health educational programs on the health, economic, and environmental advantages of balanced diets and the health risks of overconsumption and increasing reliance on meat and other animal products.

### IV. MANAGEMENT RECOMMENDATIONS FOR DECISION MAKERS

- The presently known and potential future impacts of increased circulation of Nr on human and ecosystem health and environmental quality are sufficient to warrant establishment of a quasi-permanent international research and/or research and policy assessment program. Possibilities include proposals for creating an Intergovernmental Scientific Panel on Nitrogen (ISPN) or an International Science and Policy Council on Nitrogen (ISPCN) through the United Nations (e.g., UNEP, UNESCO, FAO) or other international bodies.

- Increased attention should be given to the theoretical and practical strengths and limitations of the concept of critical loads for Nr in relation to the concept of ambient air and water quality standards for specific N compounds.
- A firm commitment is needed to long-term monitoring programs, in order to support evolving research and evaluate the effectiveness of environmental policies and programs. Chemical and biological monitoring systems should be established, and maintained or expanded in areas where they currently exist, to provide current data on the magnitude and temporal and spatial extent of emissions, transport, transformation, and deposition of Nr compounds. In addition, ecological effects monitoring networks should be established, or maintained and expanded, to track the short- and long-term effects of Nr emissions and deposition on terrestrial, freshwater, and coastal marine ecosystems.
- Coordinate action by stakeholder groups involved in N science and policy. Such coordinated action will greatly increase the effectiveness of educational, extension, and public-outreach programs by universities, government agencies, private consultants, professional societies, environmental groups, and private-sector trade and commodity associations.
- Policies to promote environmental awareness and stewardship should be strengthened. This could be done by levies or taxes, e.g., an eco-tax on products that incorporates the environmental costs related to production, transport, and waste processing, or incentives paid for adoption of improved practices that reduce Nr load. Comparative evaluations should be made of the effectiveness of education and incentive policies compared to regulation and control policies, especially for crop and animal production systems (see Figure 4).
- Wherever possible, evaluations of benefits and costs of N management methods should emphasize:

1. Practical means to internalize both the tangible benefits and the detrimental costs of increased circulation of Nr compounds in all parts of the N cascade.
  2. Multiple-pollutant/multiple-effects approaches that recognize the biogeochemical and physical linkages within the chemical climate systems and the physical climate systems of the Earth as well as among the economic, social, and environmental control systems in different regions.
  3. Such multiple-pollutant approaches also should include progressively increased awareness of linkages and interactions among the N, carbon, sulfur, phosphorus, and other nutrient cycles of the Earth.
  4. Market-based or incentive-based mechanisms should be aimed at well-defined targets that also encourage development and implementation of innovative technical and nontechnical management solutions.

- Investments in research should be focused on a balanced portfolio of objectives that include:

1. Fundamental understanding of the basic biological, chemical, and economic phenomena to be managed.
  2. Innovative approaches that derive from “out-of-the-box thinking”, especially from multidisciplinary perspectives.
  3. Further improvement of existing measures that can be adapted or modified as new knowledge and understanding become available.
  4. Ensuring that progress in dealing with one environmental problem does not lead to increased difficulties with other problems (e.g., improvements in catalytic converters to eliminate NO emissions leading to increased $N_2O$ emissions, sequestering soil carbon with increasing N additions resulting in greater $N_2O$ emissions, etc.).

- Increased investment should be made in programs fostering education of farmers and transfer of already identified

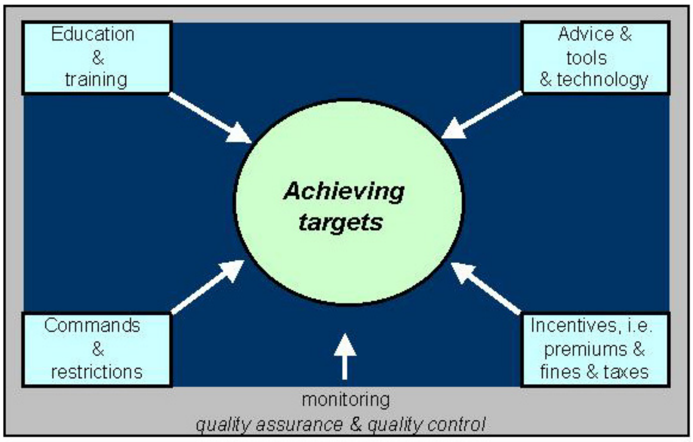

**FIGURE 4.** Framework showing tools for achieving targets in improvement of N management (from plenary presentation by O. Oenema).

improved N-management technologies in both developing and developed countries.

- Utilize N decision support systems such as NitroGenius, an interactive computer-simulation game that helps environmental managers and stakeholders: (1) understand the complexity of N-management problems, (2) learn to choose among available control measures, and (3) improve communication among stakeholders.

### V. PLANS FOR THE THIRD INTERNATIONAL NITROGEN CONFERENCE

The Second International Nitrogen Conference was designed to facilitate communications among all stakeholders in the global "nitrogen community". The Conference participants' goal in the years and decades ahead is to help nations make optimal choices about N management in food production, energy production and use, and environmental protection.

Actions must be taken and the dialogue among stakeholders must continue. Towards that end, the Third International Nitrogen Conference will be held in Nanjing, People's Republic of China, in October 2004 under the sponsorship of the Chinese Academy of Sciences and the Soil Science Society of China. The first announcement of the Conference will be issued in October 2002.

It is fitting that the Third Conference be held in Asia. The population of Asia consumes about 60% of the world's fertilizer and a growing percentage of the world's fossil fuels. These percentages will increase in the future as populations grow and per capita resource use increases. The importance of issues related to N will continue to increase in developed nations. But, in many ways, the future issues involving N management will be centered on the Asian continent.

### VI. PUBLICATIONS FROM THE SECOND INTERNATIONAL NITROGEN CONFERENCE

Cowling, E.B., Galloway, J.N., Furiness, C.S., Barber, M.C., Bresser, T., Cassman, K., Erisman, J.W., Haeuber, R., Howarth, R.W., Melillo, J., Moomaw, W., Mosier, A., Sanders, K., Seitzinger, S., Smeulders, S., Socolow, R., Walters, D., West, F., and Zhu, Z. (2001) *Optimizing Nitrogen Management in Food and Energy Production and Environmental Protection*: *Summary Statement from the Second International Nitrogen Conference, October 14-18, 2001*. Ecological Society of America, Washington, D.C. 17 pp.

Ecological Society of America. (2001) Program and Abstracts for the 2nd International Nitrogen Conference. http://esa.sdsc.edu/n2001

Galloway, J.N., Cowling, E.B., Erisman, J.W., Wisniewski, J., and Jordan, C., Eds. (2001) *Optimizing Nitrogen Management in Food and Energy Production and Environmental Protection*. Proceedings of the 2nd International Nitrogen Conference, Potomac, MD, 14–18 October 2001 (Electronic Version). TheScientificWorld, Oxford, U.K./Boynton Beach, FL. Reprinted from *TheScientificWorld* (2001) Volume 1. http://thescientificworld.com/

Galloway, J.N., Cowling, E.B., Erisman, J.W., Wisniewski, J., and Jordan, C., Eds. (2002) *Optimizing Nitrogen Management in Food and Energy Production and Environmental Protection.* Proceedings of the 2nd International Nitrogen Conference, Potomac, MD, 14–18 October 2001. A.A. Balkema Publishers, Lisse/Abingdon/Exton, PA/Tokyo.

Galloway, J.N., Cowling, E.B., Seitzinger, S.P., and Socolow, R., Eds. (2002) Proceedings of the 2nd International Nitrogen Conference, Potomac, MD, 14–18 October 2001. *Ambio* **31**.

| | | |
|---|---|---|
| Received: | November | 21, 2001 |
| Revised: | November | 27, 2001 |
| Accepted: | November | 27, 2001 |
| Published: | December | 20, 2001 |
